# Supplementary material for: Effects of Different Hamstring Eccentric Exercise Programs on Preventing Lower Extremity Injuries: A Systematic Review and Meta-Analysis
Source: Int J Environ Res Public Health. 2023 Jan 23;20(3):2057. doi: 10.3390/ijerph20032057 (PMC9916392; doi:10.3390/ijerph20032057)
Supplement: Supplementary file 1 [file ijerph-20-02057-s001.zip › Table S1ú║Basic Information of the Included Studies.pdf]

Table S1 Basic Information of the Included Studies

study	sport	sport level	Location	Sex	Age	Compliance（%）	Frequency per week	Period	Injury type	type of intervention group
Petersen.et al	football	elite&amateur	Danish	male	23，23.5	91	＞2	10weeks	hamstring injury	NHE
Gabbe.et al	football	amateur	Australia	male	23.4，23.9	46.80	＜1	12weeks	hamstring injury	NHE
Horst.et al	football	amateur	Netherland	male	24.5，24.6	91	≈2	13weeks	hamstring injury	NHE
Espinosa.et al	football	elite	Spain	famale	21	80（IG）<br>76（CG）	1	21weeks	hamstring injury	comprehensive warmup(incl NHE)
Hasebe.et al	football	youth	Japan	male	16.7/16.3	88	≈1	27weeks	hamstring injury	NHE
Askling.et al	football	elite	Sweden	male	24/26	NOT REPORT	≈2	10weeks	hamstring injury	NHE
Soligard.et al	football	youth	Norway	famale	13-17	77	＞2	1season	lower extremity injury	warm-up exercise（incl NHE）
Engebretsen.et al	football	elite	Norway	male	17-35	21	＞2	10weeks	lower extremity injury	Regular training and NHE
Granelli.et al	football	elite	USA	male	18-25	100	3	1season（5months）	the whole body	FIFA 11+ (incl NHE)
Beijsterveldt.et al	football	youth	Netherland	male	18-40	72	＞2	33 weeks	the whole body	FIFA 11 (incl NHE)
Longo.et al	Basketball	elite	Italy	male	11-21/12-24	100	≈1	9months	the whole body	FIFA 11+ (incl NHE)
Slauterbeck.et al	football，basketball, lacrosse	youth	New England	Male female	NOT REPORT	NOT REPORT	1	1year	lower extremity injury	FIFA 11+ (incl NHE)
Steffen.et al	football	youth	norway	female	13-17	52	1	5.5months	the whole body	FIFA
AI Attar.et al	football	amateur	Saudi Arabia	male	31.6	NOT REPORT	2	1season	the whole body	FIFA 11 +(incl NHE)
Granelli.et al	football	elite	USA	male	18-25	100	2-3	1season	the whole body	FIFA11+(inclNHE)
Hammes.et al	football	elite	Germany	male	45.2，43.1	98	1	9months	the whole body	FIFA 11+
Nuhu.et al	football	elite	Rwanda	male	19.9，19.7	75	3	7months	the whole body	FIFA11+(inclNHE)
Lopes.et al	football	amateur	Coimbra	male	27.7，25.8	NOT REPORT	2	20weeks	the whole body	FIFA11+(inclNHE)
Owoeye.et al	football	youth	Nigeria	male	14-19	60	＞2	6months	the whole body	FIFA11+(inclNHE)
Sebelien et al	Football	elite	Norway	male	18–39	NOT REPORT	3	1 season	hamstring injury	Warm up; NHE
Gilchrist.et al	Football	elite	USA	female	19.88	NOT REPORT	3	12weeks	ACL	INT(NHE)
Olsen.et al	handball	elite	Norway	Male female	15-17	NOT REPORT	＞1	8months	the whole body	warm up; NHE
Zarei.et al	football	youth	Iran	male	7-14	100	2	9months	the whole body	FIFA11+kids

Abbreviations:NHE, Nordic hamstring exercise; IG, intervention group; CG, control group;ACL, Anterior cruciate ligament of knee
