# Supplementary material for: Effects of Different Hamstring Eccentric Exercise Programs on Preventing Lower Extremity Injuries: A Systematic Review and Meta-Analysis
Source: Int J Environ Res Public Health. 2023 Jan 23;20(3):2057. doi: 10.3390/ijerph20032057 (PMC9916392; doi:10.3390/ijerph20032057)
Supplement: Supplementary file 1 [file ijerph-20-02057-s001.zip › Table S2ú║The results of the GRADE system.pdf]

Hamstring Eccentric Exercise vs Normal warm-up training

Patient or population: Youth and Adults
Settings: Hamstring Eccentric Exercise compared to normal warm-up training in injury prevention
Intervention: Hamstring Eccentric Exercise
Comparison: Normal warm-up training

Outcomes	Illustrative comparative risks* (95% CI)		Relative effect (95% CI)	No of Participants (studies)	Quality of the evidence (GRADE)
	Assumed risk	Corresponding risk			
	**Normal warm-up training**	**Hamstring Eccentric Exercise**			
**Lower Extremity**<br>Follow-up: 10-52 weeks	**Study population**		**RR 0.72**<br>(0.60 to 0.85)	14721<br>(15 studies)	⊕⊕⊝⊝<br>**low**[1,2,3]
	**232 per 1000**	**167 per 1000**<br>(139 to 197)			
	**Moderate**				
**Hamstring Injury**<br>Follow-up: 10-52 weeks	**Study population**		**RR 0.54**<br>(0.38 to 0.77)	6797<br>(13 studies)	⊕⊕⊕⊝<br>**moderate**[1,3,4]
	**67 per 1000**	**36 per 1000**<br>(26 to 52)			
	**Moderate**				
**Hip/Groin Injury**<br>Follow-up: 10-52 weeks	**Study population**		**RR 0.73**<br>(0.58 to 0.91)	10315<br>(10 studies)	⊕⊕⊕⊕<br>**high**[1,3]
	**37 per 1000**	**27 per 1000**<br>(21 to 33)			
	**Moderate**				
**Knee Injury**<br>Follow-up: 10-52 weeks	**Study population**		**RR 0.66**<br>(0.52 to 0.84)	13709<br>(17 studies)	⊕⊕⊕⊝<br>**moderate**[1,3,4]
	**73 per 1000**	**48 per 1000**<br>(38 to 62)			
	**Moderate**				
**Ankle Injury**<br>Follow-up: 10-52 weeks	**Study population**		**RR 0.78**<br>(0.65 to 0.93)	16365<br>(16 studies)	⊕⊕⊕⊕<br>**high**[1,3]
	**75 per 1000**	**59 per 1000**<br>(49 to 70)			
	**Moderate**				

*The basis for the **assumed risk** (e.g. the median control group risk across studies) is provided in footnotes. The **corresponding risk** (and its 95% confidence interval) is based on the assumed risk in the comparison group and the **relative effect** of the intervention (and its 95% CI).

CI: Confidence interval; **RR:** Risk ratio;

GRADE Working Group grades of evidence
High quality: Further research is very unlikely to change our confidence in the estimate of effect.
Moderate quality: Further research is likely to have an important impact on our confidence in the estimate of effect and may change the estimate.
Low quality: Further research is very likely to have an important impact on our confidence in the estimate of effect and is likely to change the estimate.
Very low quality: We are very uncertain about the estimate.

[1] There was no blinding for participants and personnel in some included studies
[2] There was high heterogeneity between studiesï¼ˆ IÂ²ï¼ž 75%ï¼‰
[3] Study demonstrated significant intervention efficacy and consistent results
[4] There was heterogeneity between studiesï¼ˆ 50% â‰¤IÂ²â‰¤ 75%ï¼‰
