# Supplementary material for: Effects of Different Hamstring Eccentric Exercise Programs on Preventing Lower Extremity Injuries: A Systematic Review and Meta-Analysis
Source: Int J Environ Res Public Health. 2023 Jan 23;20(3):2057. doi: 10.3390/ijerph20032057 (PMC9916392; doi:10.3390/ijerph20032057)
Supplement: Supplementary file 1 [file ijerph-20-02057-s001.zip › Table S3ú║Literature search words.pdf]

Literature Search

Pubmed

#1 ((((((((((((eccentric exercise[Title/Abstract]) OR (NHE[Title/Abstract])) OR (Nordic hamstring exercise[Title/Abstract])) OR (Russian leg curl[Title/Abstract])) OR (kneeling Russian hamstring curl[Title/Abstract])) OR (natural hamstring curl[Title/Abstract])) OR (bodyweight hamstring curl[Title/Abstract])) OR (FIFA11+[Title/Abstract])) OR (FIFA11[Title/Abstract])) OR (F-MARC[Title/Abstract])) OR (The 11+[Title/Abstract])) OR (warm up program[Title/Abstract])) OR (Injury prevention program[Title/Abstract])) OR (neuromuscular training[Title/Abstract])

#2 ((sport injuries[Title/Abstract]) OR (athletic Injuries[Title/Abstract])) OR (lower extremity injuries[Title/Abstract])

#3 #1 AND #2

#4 ((((randomized controlled trial[Title/Abstract]) OR (controlled clinical trial[Title/Abstract])) OR (randomized[Title/Abstract])) OR (placebo[Title/Abstract])) OR (randomly[Title/Abstract])

#5 #3 AND4

Web of Science

#1 TS=(eccentric exercise) OR TS=(NHE) OR TS=(Nordic hamstring exercise) OR TS=(Russian leg curl) OR TS=(kneeling Russian hamstring curl) OR TS=(natural hamstring curl) OR TS=(bodyweight hamstring curl) OR TS=(FIFA11+) OR TS=(FIFA11) OR TS=(F-MARC) OR TS=(The 11+) OR TS=(warm up program) OR TS=(Injury prevention program) OR TS=(neuromuscular training)

#2 TS=(lower extremity injuries) OR TS=(sport injuries) OR TS=(athletic Injuries)

#3 #1 AND #2

controlled clinical trial #4 TS=(randomized controlled trial) OR TS=(randomized) OR TS=(placebo) OR TS=(randomly) OR TS=(controlled clinical trial)

#5 #3 AND #4

Embase

#1 'eccentric exercise'/exp OR 'eccentric exercise' OR (eccentric AND ('exercise'/exp OR exercise)) OR nhe:ab,ti OR 'nordic hamstring exercise':ab,ti OR 'russian leg curl':ab,ti OR 'kneeling russian hamstring curl':ab,ti OR 'natural hamstring curl':ab,ti OR 'bodyweight hamstring curl':ab,ti OR fifa11+:ab,ti OR fifa11:ab,ti OR 'f marc':ab,ti OR 'the 11+':ab,ti OR 'warm up program':ab,ti OR 'injury prevention program':ab,ti OR 'neuromuscular training':ab,ti

#2 'lower extremity injuries' OR (lower AND ('extremity'/exp OR extremity) AND ('injuries'/exp OR injuries)) OR 'sport injuries':ab,ti OR 'athletic injuries':ab,ti

#3 #1 AND #2
